# A pilot randomized controlled trial of a stepped care intervention package for depression in primary care in Nigeria

**DOI:** 10.1186/s12888-015-0483-0

**Published:** 2015-05-01

**Authors:** Bibilola D Oladeji, Lola Kola, Taiwo Abiona, Alan A Montgomery, Ricardo Araya, Oye Gureje

**Affiliations:** 1 Department of Psychiatry, College of Medicine, University of Ibadan, Ibadan, Nigeria; 2 Department of Community Medicine, College of Medicine, University of Ibadan, Ibadan, Nigeria; 3 Nottingham Clinical Trials Unit, The University of Nottingham Queens Medical Centre, Nottingham, UK; 4 Centre for Global Mental Health, London School of Hygiene and Tropical Medicine, London, UK

**Keywords:** Depression, Primary care, Clinical trial, Stepped care intervention

## Abstract

**Background:**

Depression is common in primary care and is often unrecognized and untreated. Studies are needed to demonstrate the feasibility of implementing evidence-based depression care provided by primary health care workers (PHCWs) in sub-Saharan Africa. We carried out a pilot two-parallel arm cluster randomized controlled trial of a package of care for depression in primary care.

**Methods:**

Six primary health care centers (PHCC) in two Local Government Areas of Oyo State, South West Nigeria were randomized into 3 intervention and 3 control clinics. Three PHCWs were selected for training from each of the participating clinics. The PHCWs from the intervention clinics were trained to deliver a manualized multicomponent stepped care intervention package for depression consisting of psychoeducation, activity scheduling, problem solving treatment and medication for severe depression. Providers from the control clinics delivered care as usual, enhanced by a refresher training on depression diagnosis and management. Outcome measures Patient’s Health Questionnaire (PHQ-9), WHO quality of Life instrument (WHOQOL-Bref) and the WHO disability assessment schedule (WHODAS) were administered in the participants’ home at baseline, 3 and 6 months.

**Results:**

About 98% of the consecutive attendees to the clinics agreed to have the screening interview. Of those screened, 284 (22.7%) were positive (PHQ-9 score ≥ 8) and 234 gave consent for inclusion in the study: 165 from intervention and 69 from control clinics. The rates of eligible and consenting participants were similar in the control and intervention arms. In all 85.9% (92.8% in intervention and 83% in control) of the participants were successfully administered outcome assessments at 6 months. The PHCWs had little difficulty in delivering the intervention package. At 6 months follow up, depression symptoms had improved in 73.0% from the intervention arm compared to 51.6% control. Compared to the mean scores at baseline, there was improvement in the mean scores on all outcome measures in both arms at six months.

**Conclusion:**

The results provide support for the feasibility of conducting a fully-powered randomized study in this setting and suggest that the instruments used may have the potential to detect differences between the arms.

**Trial registration number:**

ISRCTN46754188 (ISRTCN registry at isrtcn.com); registered 23 September 2013, details of the pilot study added 12/02/2015.

## Background

Depression is a common problem in primary care and is often a prominent cause of unmet need for mental health care. In Nigeria, studies report prevalence estimates in the range of 10-20% in primary care [1,2]. However primary care providers often do not have the expertise to diagnose and manage depressed patients. In a WHO multi-centre study of mental illness in general health care, in which Nigeria was a participant, less than half of patients with mental disorders identified by the research diagnostic interview were detected by primary care physicians [3]. In most low and middle income countries, the bulk of primary health care is provided by non-physician primary health care workers. Even though figures are not available on the rates of mental illness identified by these primary care workers, it is likely that it might be lower than that for physicians.

Some critics have raised concerns about the cross-cultural validity of mental health diagnosis such as depression [4] however, cross-national studies consistently support the presence of depression as diagnosed using standard diagnostic instruments and criteria across cultures [5]. The validity of depression diagnosis across cultures and specifically in Nigeria is further supported by the correlations between depression severity and disability, in keeping with findings from other cultures [6-8].

Nigeria like many low and middle income countries has inadequate specialist mental health personnel, with less than one psychiatrist to one million population and with the few available specialists inequitably concentrated in urban settings [9]. This lack of mental health human resources is one of the major factors contributing to the large treatment gap for mental disorders in Low and Middle income countries (LMICs) [10], which often exceeds 75% of those suffering from mental disorders in these countries. It has been suggested that the most efficient and effective way to reduce this gap in resource-constrained settings is to integrate mental health into primary health care [11]. There is evidence that a collaborative stepped care approach in which some tasks are performed by primary care providers offers the most effective way to implement this integration [12].

A stepped care approach involves the provision of different levels of treatment intensity with the most intensive treatment reserved for the more severe cases. Task shifting involves non-specialist health workers delivering most of the frontline care while specialists only provide ongoing training, supervision and support as well as care for the most severe cases [13]. Studies from LMIC of Asia and Latin America and a few recent studies from Africa suggest that effective low cost, low intensity treatments can be administered by lay or minimally trained primary health care workers. For example, in Chile, the effectiveness of a stepped-care programme was compared with usual care in primary-care management of depression among poor women in Santiago, Chile [14]. The interventions were delivered mostly by non-medical primary care workers; the study reported a large and significant improvement in the outcome measures of patients in the stepped-care programme compared with usual care.

A pilot study of task shifting in primary care in Zimbabwe demonstrated that it was feasible for lay workers to deliver an intervention for common mental disorders based on problem solving treatment (PST). The treatment was reported to be acceptable to the community and was efficacious in reducing psychological morbidity amongst the participants [15]. Problem solving treatment is easily learned and acceptable to patients. A randomized control trial in the UK showed that in treating depression in primary care, PST is as effective as amitriptyline, feasible, and acceptable to patients [16].

Even though there is evidence that stepped care programs with task shifting embedded improve depression outcomes, the evidence for its applicability and effectiveness is still very sparse. In particular, there is a need for empirical information on how to effectively design, plan, and deliver these interventions in primary care settings characterized by extreme resource constraints, both human and material, as it exists in most of Sub-Saharan Africa. The aim of this study is to design and test the feasibility of a randomized controlled trial of a stepped care intervention package for depression in primary care, consisting of psychological and pharmacological treatment approaches delivered by non-physician primary care providers with support and supervision by physicians and psychiatrists.

## Methods

### Study design and setting

This is a pilot two-arm parallel cluster randomized control trial of a manualized stepped care intervention for depression. The cluster design was favoured to reduce contamination within clinics. The unit for randomization was the primary health care center.

The study was carried out in six primary health care centers (PHCC), randomly selected from two local government areas (LGA), one rural and the other urban, in Oyo State. Oyo State is one of the six states in the Southwest geopolitical zone of Nigeria with a population of about 4.5 million.

In Oyo State, primary care service is mainly delivered by non-physician primary care providers consisting of nurses, community health officers and community health extension workers. Each of these categories of providers has a minimum of three years post-secondary education and are certified by their respective boards. Supervision for all the clinics in each LGA is provided by one general practitioner employed by the government and designated as the Primary Health Care Coordinator for the Local Government.

A listing of all eligible PHCCs was obtained (eligible clinics were those with a full complement of primary care workers and provide a broad range of clinical service). From this listing, 3 clinics were randomly allocated to the intervention arm and 3 to the control arm. The allocations were done by an independent statistician using a table of random numbers.

Ethical approval was obtained from the University of Ibadan/University College Hospital Joint Ethical Review Committee.

### Training of providers and research staff

The Primary Health Care Coordinators in each of the selected LGAs and the Matrons in charge of each of the selected clinics were approached and briefed about the study. Following this, 3 health care providers from each clinic nominated by the matrons and doctors were invited for training. In total, 18 primary health care providers were trained, this included- 6 nurses, 3 community health officers and 9 community health extension workers.

The primary health care workers (PHCWs) from the intervention clinics had an initial 3-day training. The training focused on the identification and treatment of depression. They received training on how to manage depression using psychoeducation, activity scheduling and problem solving treatment (see below for description of intervention). They had a further 3-day top-up training about a month into the study to reinforce the treatment modalities they had been trained in and to identify any difficulties they had in administering the treatments. The training consisted in didactic lectures enhanced with clinical demonstrations and role playing exercises.

The PHCWs from the control clinics received 2 days of training on identification and standard treatment of depression. They were also provided with manuals detailing the diagnosis and treatment for depression. However the choice of intervention was at the discretion of the health care provider. Hence patients from the control clinics received treatment as usual enhanced by this refresher training on depression given to the PHCWs selected from the participating clinics.

Lay research assistants with at least a college degree were recruited and trained to administer the screening instruments and conduct the outcome assessments.

All the training was delivered by 2 psychiatrists (BD and OG).

### Participants and recruitment

**Figure 1:**
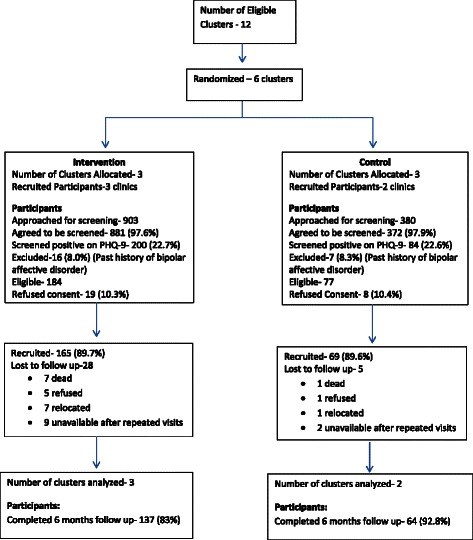
Flow diagram of recruitment into the study.

Consecutive adult attendees (aged 18 years and over) presenting to the selected clinics were individually approached in the waiting area of the clinic by the research assistants. The study protocol was explained and those who consented were administered the screening instruments to determine their eligibility for inclusion into the study. Screening was conducted with the Patient Health Questionnaire (PHQ-9), previously validated in the setting of the study [17,18], and a set of questions derived from the Composite International Diagnostic Interview (CIDI). These questions were to establish whether patients met diagnostic criteria for DSM-IV major depression and to exclude a diagnosis of bipolar disorder or the presence of psychotic symptoms. Patients who scored 8 or more on PHQ-9, did not have bipolar disorder or psychosis, were not currently undergoing treatment for a mental illness and who provided informed consent were recruited into the study (Figure 1). Other exclusion criteria included serious suicidal ideation or attempts in the previous week, presence of a serious physical illness requiring emergency attention, and participants who indicated they were not going to be available for follow-up for the duration of the study.

Information about place of residence and contact phone number was obtained from eligible and consenting respondents. They were then handed their PHQ-9 scores and directed to see one of the 3 trained primary health care workers. The PHCW using an adapted version of the WHO mhGAP guide confirmed the diagnosis of depression and determined the intervention to administer. All the patients who screened positive on PHQ-9 received a diagnosis of depression from the PHCWs. Patients in the intervention clinics were offered treatment based on a manualized stepped care intervention package (described below).

### Baseline and outcome measures

Baseline and outcome measures were administered by trained research assistants at the patients’ homes. These research assistants were blinded to patient allocation into intervention or control arms. The baseline measures were administered within 72 hours of screening. The measures consisted of the WHO Disability Assessment Schedule 2.0 (WHO-DAS II ) [19], WHO quality of life scale short form (WHOQOL-Bref) [20], and the Service Utilization Questionnaire (SUQ) [21].

The WHODAS was developed for measuring functioning and disability in accordance with the International Classification of Functioning, Disability and Health across different populations. The WHODAS II has high internal consistency (Cronbach’s alpha, α: 0.86), a stable factor structure; high test-retest reliability (intraclass correlation coefficient: 0.98); and good concurrent validity in patient classification [19]. The WHOQOL-Bref is a cross-cultural tool for subjective evaluation of health related quality of life; it has excellent internal reliability (Cronbach’s alpha 0.86) [22]. The SUQ is derived from the Client Service Receipt Inventory (CSRI). The CSRI is designed to collect information about the use and costs of health and social services and other economic impacts such as time of work due to illness [23,24]. These tools have been previously used by us in the setting of the study [8,25].

Follow up assessments on participants were conducted at 3 and 6 months and consisted of all measures administered at baseline as well as the PHQ-9. The outcome measures assessed were: improvement in depression at 6 months follow up; (improvement was defined as at least a 50% reduction in baseline score or a score of 5 or less on the PHQ-9 [17,26,27]), changes in WHOQOL, WHODAS and SUQ scores. Other important parameters to determine the feasibility of the full trial included the number of participants successfully followed up as well as participants’ adherence to interventions (assessed by setting projected progress indicators- see below). Important outcomes were: recruitment rates, the ability of the PHCW to incorporate depression care into their routine practice and their adherence to intervention guidelines.

### The stepped-care intervention

**Figure 2:**
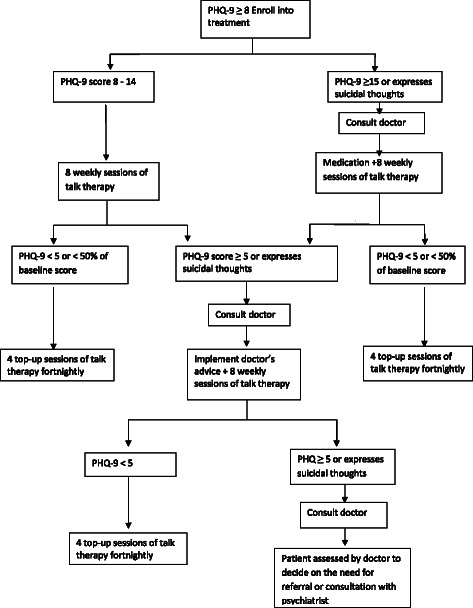
Treatment flow chart.

A stepped care intervention package for depression was provided for the patients in the intervention clinics while those in the control clinics had enhanced care as usual. In the stepped-care intervention package, the treatment offered was determined by a patient’s score on the PHQ-9 (See Figure 2). The package consisted of psychoeducation, activity scheduling, and an adapted form of problem solving treatment as well as antidepressant medication for those more severely ill or not responding to other treatments. All interventions were carried out in the Yoruba language by health care providers fluent in the Yoruba language and experienced in practicing in the locality. The Yoruba translations were done by panels of bilingual experts using standard protocols of iterative back translation.

The interventions were adapted to the local language and cultural context while preserving their core elements [28]. The process of adaption involved an initial series of meetings and focus group discussions with health care providers experienced in working in primary care and with knowledge of the local culture, beliefs and practices, to discuss the chosen interventions as well as in-depth interviews with patients. Insight gained from these interactions informed adaptation in terms of appropriate language and local terminologies that would be more acceptable in the cultural context. For example, the word ‘problem’ was replaced by a term better interpreted as ‘challenge’ or ‘difficulty’. In psychoeducation, the use of the label ‘mental disorder’ was avoided in describing depression to reduce stigma, it was rather presented as a medical problem that could be treated. It was also agreed that tasks that require literacy (reading and writing) be minimized and the use of pictoral charts for rating mood and describing the intervention process be used in the patient information booklets. The activity scheduling and PST were tailored to address more culturally appropriate tasks as applicable to individual patients.

To facilitate consultation with the supervising doctors, each of the trained PHCWs in the intervention clinics was provided with a mobile phone. The supervising doctors were also provided with mobile phones. These mobile phone numbers were linked in a closed user group network where calls within the network were free. The doctor similarly had access to the psychiatrist for consultation for difficult cases. Ongoing support and supervision for the PHCW delivering the interventions were provided by the team. A member of the team scheduled visits to sit in with the providers to observe some treatment sessions as well as listened to recordings of other sessions and provided feedback to the individual PHCWs.

In psychoeducation, the diagnosis of depression is explained to the patient in simple language using local expressions while avoiding the labeling of ‘mental illness/disorder’. The patient is helped to understand that the symptoms being experienced are not as a result of laziness or supernatural forces but an ailment that is common and amenable to treatment. The patient is encouraged to ask questions and be free to express their feelings. We used a local adaptation of Problem Solving Treatment for Primary Care (PST-PC), [15,28,29] a seven-step common sense talk therapy that aims at helping patients solve troublesome problems that contribute to causing or prolonging the depressive episode. It includes the identification and exploration of the problems currently being faced by the patient and aiding the patient to develop and implement practicable solutions. In activity scheduling, patients are encouraged to carry out more activities that are important or pleasurable to them.

In this study, amitriptyline was the antidepressant of choice. Amitriptyline is the only antidepressant medication currently listed in the standing order (a book of instructions guiding the practice of PHCWs in the Nigerian health system). PHCWs are allowed to prescribe this medication under the supervision of a primary care physician. Should the need arise; the physicians could prescribe any other antidepressant medication to patients referred to them following the stepped care approach.

Figure 2 provides a diagrammatic sketch for intervention decisions. In the first step of the intervention package, all patients with a PHQ-9 score of between 8 and 14 receive 8 weekly sessions of individual talking therapy delivered by the PHCW. For participants whose PHQ-9 score is 15 or more at the outset or who express suicidal ideation, the PHCW consults with the doctor on phone immediately. The doctor decides whether to see and review the patient or gives instruction on the prescription of amitriptyline to the patient. Participants who are prescribed antidepressant medication nevertheless also receive weekly sessions of talking therapy in addition to the antidepressant medication.

Following completion of the 8 weeks of treatment, the PHCW administers the PHQ-9 to assess level of improvement and decides on interventions for the second step. Participants who improve, indicated by a PHQ-9 score of 5 or lower or less than half of baseline score, receive four fortnightly top up talking therapy sessions over an additional 8 weeks. Those who do not improve are reviewed by the doctor and considered for medication, if none has been prescribed in the earlier step or medication is reviewed if already on antidepressants. Such participants are also offered additional weekly talking therapy sessions for 8 weeks.

### Structure of the sessions

Session 1- The patient hands over the screening sheet containing the PHQ-9 score and presenting symptoms. The primary care worker then proceeds to confirm the diagnosis of depression. This first session is mainly focused on developing rapport, laying the foundation for a therapeutic relationship with the patient as well as delivering psychoeducational material.

Session 2- PHCW re-emphasizes the key points in psychoeducation and commences activity scheduling. Patient is assisted to make a list of pleasurable activities and household chores that they have stopped doing; the PHCW then works with the patient to select a few to be carried out over the next week.

Session 3- PHCW reviews with the patient the activities carried out over the last week, and emphasizes psychoeducation as well as the importance of the therapy sessions and activities to alleviate symptoms of depression. In this session, PST is introduced to the patient and PHCW works with patient to identify problems that they are currently faced with. Other activities are then scheduled.

Session 4- PHCW explores problems identified from the last session and works with patient to prioritize and select a specific problem to work on. The patient is encouraged to brainstorm practical and feasible solutions to this problem and then to think over which solution to try first. In addition, more pleasurable activities are scheduled.

Session 5- PHCW reviews with patient the activities scheduled as well as the attempts at problem solving. If the previous problem was successfully resolved, another is selected and the problem solving process is repeated. Otherwise the barriers or difficulties to solving the problem are reviewed and alternative solutions sought.

Subsequent sessions follow a similar pattern to session 5. The PHCW always reviews with patients their attempts at problem solving, praising their efforts and relating improvement in depression symptoms to their improved ability to deal with problems.

In this study, sessions were scheduled at times agreed to by both the patient and the PHCW usually outside of busy clinic hours. This ensured that sessions could be conducted in strict privacy and with less likelihood of intrusion. Each session lasted between 25–45 minutes, with an average duration of 35 minutes.

We designed a fidelity assessment questionnaire based on the tasks and intervention for each treatment session. This questionnaire was scored by trained research assistants on a 4 point likert scale to assess the competence of the PHCW in delivering the interventions and adherence to the intervention protocol as detailed in the manual.

### Process indicators for the intervention arm

We set projected benchmarks for the intervention clinics as a means of monitoring the process of service delivery (See Table 1). We projected that at least 95% of patients referred to the primary care provider would complete the first session of the intervention package. Other benchmarks included were: 20% of participants will require antidepressant medications, 50% of patients should complete 6 psychological intervention sessions, about 15% of patients would be seen by the primary care physician and 5% would require referral to a psychiatrist.

**Table 1:** **Process indicators for the intervention arm**

Process indicators	Number	Percentage	Projected benchmark
Proportion of patients who received at least the first psychoeducation session	165 of 165	100%	95%
Proportion of cases who received at least 2 sessions of talking therapy?	123 of 165	74.5%	70%
Proportion of patients who completed at least 6 sessions of talking therapy	57 of 165	34.5%	50%
Proportion of cases who received antidepressants	42 of 165	25.5%	20%
Proportion of patients receiving antidepressants who completed at least 3 months treatment	25 of 42	60%	55%
Proportion of patients for whom telephone contact to doctor was made	48 of 165	29%	30%
Proportion of patients referred to the doctor	17 of 165	10.3%	15%
Proportion of patients referred to psychiatrist	3 of 165	2%	5%

### Data analysis

We used appropriate descriptive statistics to summarize indicators of feasibility such as recruitment, amount of intervention received, and follow up. Similarly, we describe baseline characteristics of trial participants, and outcome measures at six months follow up. Linear regression models taking into account clustering effects were used to estimate between-group differences in outcome measures. All analysis were conducted using Stata 13.0.

## Results

A total of 1283 consecutive attendees were approached for screening, while 1253 agreed to be screened (representing 97.7% of those approached) (Figure 1). The rate of participants refusing to be screened was not different in the control and intervention clinics (2.1% and 2.4% respectively). Of those screened, 284 (22.7%) were positive on PHQ-9 (that is, scored ≥ 8) (22.7% in intervention and 22.6% in the control arms). Following assessment with questions derived from the CIDI, 23 of screen positives did not satisfy inclusion criteria and were excluded, leaving 261 (or 20.8% of screened sample). These 23 were excluded for saying yes to either of the two questions screening for bipolar affective disorder (either a period of four days or more when they were unusually elated, irritable or argumentative in their lifetime or have ever been treated for a manic episode). Of the eligible subjects, 27 (10.3%) refused consent or indicated that they would not be available through the period of follow-up. The rates of participants refusing consent were similar in both arms. Of the remaining 234 consenting patients, 165 were recruited from the intervention arm while 69 were from the control arm (Figure 1).

The imbalance between the arms in number of patients approached and recruited resulted from one of the clinics allocated to the control arm being shut down a few weeks after entering into the study.

Table 2 compares the demographic profile at baseline between the intervention and care as usual arms. The trial arms appeared to be well balanced in regard to their level of depression at baseline, offering reassurance that there was no major selection bias operating.

**Table 2:** **Characteristics of trial arms at baseline**

	Intervention	Control
Number of clusters	3	3
Number of individuals	165	69
Mean age, sd (years)	43.2 (15.3)	43.1 (18.9)
Mean years of education (s.d)	8.2 (5.2)	8.2 (5.4)
Sex, % female	80.6	79.7
Mean PHQ9 score, sd	11.3 (3.5)	11.3 (3.9)

### Process indicators for the intervention arm

The process indicators are presented in Table 1. All the participants received the first intervention session, up to 75% had at least two sessions, this was slightly above our projected benchmark of 95% and 70% respectively. About 25% of participants were prescribed antidepressants, out of which 60% of them completed 3 months of treatment. Only 10.3% of patients seen required consultation with the primary care physician and only 2% were referred for consultation with a psychiatrist.

The providers made calls to the physicians to discuss 48 patients (29% of participants receiving intervention) while, only a third of these (17 patients, 10% of the patients receiving intervention) needed face-to-face consultations with the doctor.

### Feedback from health care providers

Health care providers were encouraged to provide feedback throughout the duration of the study to enable us identify any difficulties and modifications that needed to be made to the protocol for the full trial. We used a mixed method approach to obtain feedback from health care providers. During the top-up training, providers were administered a combination of open and close ended questionnaires and we conducted in-depth interviews with providers. The health care providers found the training useful to their practice, it improved their ability to identify mental health problems and improved their confidence in managing patients presenting with psychological problems. The providers generally had little difficulty in delivering the psychoeducation and PST. Recorded sessions also showed moderate to good overall adherence to the manualized intervention programme.

The main problems identified by the providers in the initial phase of the study included: patients poor compliance with follow up appointments; providers often had difficulty making out time to attend to the patients during busy clinic hours; and the doctors were sometimes unable to attend to telephone calls when they were needed due to other clinical commitments.

The most common reasons for defaulting from scheduled visits were: a) patient reports of being well and no longer in need of treatment; b) patient expects medication to be prescribed, feeling that the talking therapy was not a sufficient reason for repeated clinic visits; c) inability to afford the cost of transportation to clinic; and d) concerns about whether medication may lead to dependence.

Some of these difficulties were addressed during the top-up training and the following modifications were made:

- Primary care providers were told to emphasize to the patients the importance of the talking therapy sessions, the need for compliance and to link improvement in symptoms to the sessions.
- Participants who were in need would be provided with a small amount of money at each visit to offset their transportation costs to keep their follow up appointments.
- Providers were encouraged to make calls to patients as a reminder a day before their appointments were due.
- In consideration of the busy schedule of the providers, appointments for patients on the trial were to be scheduled outside of busy clinic hours but before the closing of the working day.

### Outcome measures

About 83% (137) of the participants from the intervention arm and 92.8% (64) from the control arm were successfully administered the outcome assessments at the 6 months follow up visit (Figure 1). Participants who were successfully followed-up compared with those who were not were more likely to be female (% female 81.8 vs. 75.0), older (43.3 years, s.d. 15.44 vs. 39.7 years s.d. 15.08), have fewer years of education (8.8 years s.d. 4.93 vs. 9.3, s.d. 4.53) and have slightly higher PHQ-9 score at baseline (11.5, s.d. 3.67 vs. 10.3, s.d. 2.35).

Improved depression scores (that is a score of less than 5 or at least 50% reduction in baseline scores) were observed in 73.0% of participants in the intervention clinics compared to 51.6% in the usual care group at 6 months follow up (OR 2.7; 95% CI 1.43 to 5.25).

The mean change in scores for PHQ-9, WHO-QOL and WHO-DAS for the intervention and the care as usual arms and differences in mean scores between arms along with their 95% confidence intervals are presented in Table 3.

**Table 3:** **Mean change in scores on outcome measures over six months**

		Intervention		Control		Change in scores between baseline and six months		Difference in means at 6 months intervention vs control	
	Intracluster correlation coefficients (ICC)	Baseline n = 165	6 months n = 137	Baseline n = 69	6 months n = 64	Intervention	Control	Unadjusted (95% CI)	Adjusted *(95% CI)
		Mean (SD)	Mean (SD)	Mean (SD)	Mean (SD)	Mean change (95% CI)	Mean change (95% CI)		
Depression (PHQ-9)	0.045	11.3 (3.5)	4.1 (4.4)	11.3 (3.9)	5.5 (5.2)	−7.4 (−8.3 to –6.5)	−5.9 (−7.4 to –4.4)	−1.4 (−4.3 to 1.5)	−1.4 (−2.6 to 1.2)
WHODAS	0.042	21.0 (7.0)	15.9 (5.4)	21.6 (6.4)	16.3 (6.9)	−5.6 (−6.9 to 4.3)	−5.5 (−7.3 to –3.7)	−0.5 (− 4.6 to 3.7)	−0.4 (−3.8 to 2.9)
WHOQOL	0.049	73.6 (13.5)	85.5 (12.9)	68.3 (13.6)	78.2 (11.5)	12.5 (9.7 to 15.4)	11.0 (7.7 to 14.3)	7.3 (2.8 to 11.9)	5.2 (−0.5 to 11.0)

*Adjusted for baseline scores, age and sex.

WHODAS- WHO disability assessment schedule. WHOQOL- WHO quality of life instrument. SD- standard deviation. CI- confidence interval.

Patients in the intervention arm had a more favourable assessment of the care they received. Compared to baseline, at six months follow-up, fewer patients in the intervention arm judged their service use was affected by a perception that 1) the providers were less knowledgeable or responsive to their health needs (20.1% vs. 1.5%), or 2) the treatment they received was not good enough (20.9% vs. 3.7%). No such changes in reported experience of service were observed among the patients in the control arm.

Although there was some suggestion of benefit among the intervention arm on the chosen outcome measures, this pilot study was not designed with sufficient power to detect differences in outcome measures between the intervention and care as usual groups. However, the improvements in the scores suggest that these instruments could be useful as measures of improvement over time.

## Discussion

This study demonstrated that it would be feasible to conduct a full scale randomized controlled trial comparing our improved intervention to usual care for the treatment of depression in primary care in Nigeria. We found that primary health care workers could be trained to identify depression and deliver effective interventions for depression within the primary health care clinic setting. The results once again demonstrate the high rates of depression in primary health care in Nigeria; one out of every five patients presenting to primary health care clinics qualified for a diagnosis of depression. This finding underscores the urgent need for randomized controlled trials to test the feasibility, affordability and effectiveness of integrating services for depression into primary care in low-resource settings Our rates of recruitment and follow up over a six-month period were high; 89.7% of eligible participants were successfully enrolled into the study and 85.9% of those enrolled completed outcome assessments at six months. Scores on outcome measures showed improvement at six months follow up, there was a slight benefit among the intervention arm on the chosen outcome measures These differences in outcome measures suggest that the intervention used in this pilot has the potential to be more effective than usual care.

A key strength of this study is that we used existing human resources typically available within the primary health care setting in Nigeria to deliver the intervention. Using existing resources is one important way of ensuring sustainability of new community mental health programmes [30]. However, considering the busy schedule of most primary care clinics, coupled with inadequate staffing which may make it impractical for the primary care providers to routinely screen all patients, we used lay research assistants to conduct the initial screening for depression. Even though the screening was done by lay research assistants the primary care provider confirmed the diagnosis and determined the eligibility of the patient to receive the intervention. This was done to improve recognition of depression by primary care workers and to ensure that only eligible patients were recruited into the study. Current evidence supports the need to supplement screening questionnaires with clinical judgment in the diagnosis of depression in primary care [31]. We have also shown that it was possible for the primary health care providers to integrate depression intervention into their routine work.

A novel approach introduced in this study to enhance supervision and support for primary health care providers was the incorporation of mobile phones. To the best of our knowledge this is the first time mobile telephones were being used to facilitate depression care in Sub-Saharan Africa. Primary health care providers were enabled to make calls to the primary care physicians to get help on difficult cases as well as call their patients to remind them of their clinic sessions. As noted in the results section, providers made calls to the physicians to discuss 48 patients (29% of patients receiving intervention) while, only a third of these (about 10% of the patients receiving intervention) needed face-to-face consultations with the doctor. This is particularly useful in our primary care setting where typically one physician supervises the entire health facilities in a local government which could consist of 8–15 primary care clinics.

The main problem encountered in trying to contact the primary care physicians was that they were sometimes unable to attend to telephone calls when they were needed due to other clinical and administrative commitments. As a result of this experience, a modification was made to the program such that, other than for emergencies (e.g. necessitated by a drug reaction or evidence of suicidality) a specific time is set for the PHCWs to phone the physicians to conduct reviews. This ensured that requests for reviews did not clash with GP’s other commitments.

This study provided us with an opportunity to test the feasibility of using our manualized intervention package in primary care. While the primary care providers had little difficulty in learning and applying the interventions, default rates were high especially for patients receiving the psychological interventions alone. Only 34.5% of patients receiving PST alone completed 6 sessions whereas up to 60% of patients on antidepressant medication completed 3 months of treatment. The difficulties experienced with patient compliance have led to the development of measures to improve patient adherence rates in the design of a full trial. These measures include training the health care provider to emphasize the importance of the talking therapy sessions, linking improvement in patients’ symptoms to attendance at therapy sessions. Other measures included the use of telephone reminders to the patients and subsidizing the cost of transportation for patients who could not afford the cost of transporting themselves to the clinics for the weekly talking therapy sessions.

This study was designed to explore the feasibility, acceptability and potential effectiveness of a multi-component stepped care intervention package in primary care. As with any pilot study a limitation is our inability to assess the effectiveness of the intervention. The relative contribution of the psychological component of our intervention (Problem Solving Treatment) is impossible to assess in a study using a multi-component, stepped care intervention. Another limitation is that we could not assess whether the category of health care provider made a difference in default rates or outcome measures due the small numbers of providers in different cadres used in this study.

## Conclusions

Our pilot study demonstrated that it is possible for non-specialist primary health care providers to diagnose and deliver interventions for depression in primary health care settings in Nigeria with support and supervision from physicians and specialists and also provided information on the feasibility of conducting randomized trial of complex interventions for depression in these settings. Empowering non-physician primary care providers to deliver interventions for common mental disorders through training, on-going support and supervision from physicians and collaborations between specialist services and primary care might play a pivotal role in reducing the treatment gap for depression.

## Abbreviations

PHCW: Primary health care worker
PHCC: Primary health care center
PHQ-9: 9-item Patient Health Questionnaire
WHO: World Health Organization
LMIC: Low and middle income countries
PST: Problem solving treatment
LGA: Local government area
CIDI: Composite International Diagnostic Interview
mhGAP: WHO mental health gap action programme
WHO-DAS: World Health Organization Disability Assessment Schedule
WHOQOL: World Health Organization Quality of Life Questionnaire
SUQ: Service Utilization Questionnaire
